# High hospital research participation and improved colorectal cancer survival outcomes: a population-based study

**DOI:** 10.1136/gutjnl-2015-311308

**Published:** 2016-10-20

**Authors:** Amy Downing, Eva JA Morris, Neil Corrigan, David Sebag-Montefiore, Paul J Finan, James D Thomas, Michael Chapman, Russell Hamilton, Helen Campbell, David Cameron, Richard Kaplan, Mahesh Parmar, Richard Stephens, Matt Seymour, Walter Gregory, Peter Selby

**Affiliations:** 1 Leeds Institute of Cancer and Pathology, University of Leeds, St James's University Hospital, Leeds, UK; 2 Cancer Research UK Centre, University of Leeds, St James's University Hospital, Leeds, UK; 3 MRC Bioinformatics Centre, Leeds, UK; 4 Leeds Institute of Clinical Trials Research, University of Leeds, Leeds, UK; 5 Leeds Teaching Hospitals NHS Trust, Leeds, UK; 6 National Cancer Intelligence Network, London, UK; 7 Cancer Research UK, London, UK; 8 Department of Health, Research and Development, London, UK; 9 Clinical Research Facilities, and Cancer Research, University of Exeter Medical School, Exeter, UK; 10 NIHR Cancer Research Network, Leeds, UK; 11 Edinburgh Cancer Research Centre, University of Edinburgh, Western General Hospital, Edinburgh, UK; 12 MRC Clinical Trials Unit at University College London, London, UK; 13 NCRI Consumer Liaison Group, NIHR Cancer Research Network, Leeds, UK

**Keywords:** COLORECTAL CANCER, HEALTH SERVICE RESEARCH, CLINICAL TRIALS

## Abstract

**Objective:**

In 2001, the National Institute for Health Research Cancer Research Network (NCRN) was established, leading to a rapid increase in clinical research activity across the English NHS. Using colorectal cancer (CRC) as an example, we test the hypothesis that high, sustained hospital-level participation in interventional clinical trials improves outcomes for all patients with CRC managed in those research-intensive hospitals.

**Design:**

Data for patients diagnosed with CRC in England in 2001–2008 (n=209 968) were linked with data on accrual to NCRN CRC studies (n=30 998). Hospital Trusts were categorised by the proportion of patients accrued to interventional studies annually. Multivariable models investigated the relationship between 30-day postoperative mortality and 5-year survival and the level and duration of study participation.

**Results:**

Most of the Trusts achieving high participation were district general hospitals and the effects were not limited to cancer ‘centres of excellence’, although such centres do make substantial contributions. Patients treated in Trusts with high research participation (≥16%) in their year of diagnosis had lower postoperative mortality (p<0.001) and improved survival (p<0.001) after adjustment for casemix and hospital-level variables. The effects increased with sustained research participation, with a reduction in postoperative mortality of 1.5% (6.5%–5%, p<2.2×10^−6^) and an improvement in survival (p<10^−19^; 5-year difference: 3.8% (41.0%–44.8%)) comparing high participation for ≥4 years with 0 years.

**Conclusions:**

There is a strong independent association between survival and participation in interventional clinical studies for all patients with CRC treated in the hospital study participants. Improvement precedes and increases with the level and years of sustained participation.

**Significance of this study**

**What is already known on this subject?**

- Only a few studies have investigated whether a hospital's research activity for a specific disease is associated with better survival for all of their patients with that disease.
- Disease-specific studies in coronary artery disease and ovarian cancer suggested improved survival for patients treated at the more research-active hospitals.
- Using colorectal cancer (CRC) as an example, in >200 000 patients we test the hypothesis that high, sustained hospital-level participation in interventional clinical trials improves outcomes for all patients with CRC managed in those research-intensive hospitals.
- The ‘big dataset’ allows us to explore the possible causal link.

**What are the new findings?**

- Patients treated in hospitals with high rates of research participation (≥16%) in their year of diagnosis had lower postoperative mortality (p<0.001) and improved 5-year survival (p<0.001) after adjustment for casemix and hospital-level variables.
- The effects increased with sustained participation in research, with a reduction in postoperative mortality of 1.5% (6.5%–5%, p<10^−6^) and an improvement in 5-year survival of 3.8% (41.0%–44.8%, p<10^−19^) comparing high participation for ≥4 years with 0 years participation.
- Improvement precedes and increases with the level and years of sustained participation.
- The effects are seen across all NHS hospitals that care for patients with colorectal cancer and is not restricted to academic centres or hospitals with large practices.

**How might it impact on clinical practice in the foreseeable future?**

- Our results allow investigators to show patients, healthcare commissioners and policymakers that being treated in a hospital active in clinical research is strongly associated with better outcomes for patients with colorectal cancer. The data provide an additional incentive to integrate research into standard medical care.

## Introduction

Clinical research provides evidence to improve the care of patients in the future. It has also been asserted that patients who participate in clinical research may achieve better outcomes as a result, regardless of whether they are allocated novel or standard treatment and whether the trial subsequently delivers a positive result.^1^ ^2^ However, unquantifiable prognostic differences between patients who are or are not offered research, and do or do not consent to participate, have to date made this claim impossible to substantiate.^3–5^

A related but different question, more feasible to address, is whether the level of clinical research activity within a hospital or multidisciplinary team (MDT) correlates with the outcomes of all patients treated by that hospital or MDT.^2^ ^6–8^ If such a correlation exists, such research activity could simply be a non-causative surrogate marker of an institution's quality. On the other hand, can commitment to research participation, especially the uptake of research in previously research-inactive teams, itself drive improvements in care? The processes of adopting and participating in research might improve outcomes through diverse mechanisms, stimulating teams to consider new evidence, introduce new improved cancer treatments and equipment, better quality assure their treatments and investigations and rationalise decision-making. It is plausible that such effects, which may be stimulated by research directly involving only a minority of patients, have a bystander effect on non-research patients cared for by the same team.

The impact of research activity on healthcare performance has recently been thoroughly reviewed.^7^ ^8^ It was concluded that it is reasonable to suggest that when clinicians and hospitals engage in research, there is a likelihood of improved performance but the evidence related mainly to improved processes of care. However, a few studies have investigated whether a hospital's research activity is associated with survival for all of their patients. Population-level data for England 2005–2010 showed a small but significant reduction in any-cause inpatient mortality after acute admission among hospitals with higher research recruitment.^9^ Disease-specific studies in coronary artery disease in the USA^10^ and ovarian cancer in Germany^11^ ^12^ also found improved survival for patients treated at the more research-active hospitals. However, there is a pressing need for further large-scale research to confirm and evaluate the association and to begin to consider evidence that may allow us to distinguish between non-causative and causative links.

In 2001, the National Institute for Health Research Cancer Research Network (NCRN) was established to provide the English NHS with clinical infrastructure to improve the recruitment, speed, quality and integration of clinical cancer research in all parts of the NHS. An important goal of the NCRN was to include all types of hospital Trust. Studies within the NCRN portfolio were offered for participation across the Networks through the trials units and network managers. Recruitment centres were not selected by chief investigators alone. It rapidly changed much of the NHS to be research-intensive and greatly increased the number of studies in the national portfolio, the number of patients recruited and the number of staff involved in research.^1^ ^13^ ^14^ Over the same period, the National Cancer Data Repository (NCDR) has collated and combined existing datasets (such as cancer registrations and hospital admissions) to create comprehensive individual-patient records of cancer diagnoses, demographics, treatments and mortality.^15–19^

In this paper, we test the hypothesis that patients treated in hospitals with high rates of interventional clinical research participation have improved outcomes compared with patients treated at otherwise similar but research-inactive institutions. We focus on colorectal cancer (CRC): this is because the NHS requires all patients with CRC to be managed in hospitals with a CRC MDT, and it is rare for patients to be transferred away from their ‘home MDT’ for primary treatment. We further hypothesise that the relationship between interventional clinical research participation and outcomes is dependent upon the degree and duration of research participation.

## Methods

Information was extracted on patients diagnosed with a first primary CRC (ICD V.10^20^ code C18–20) between 1 January 2001 and 31 December 2008. Dates of diagnosis and death (to 30 June 2010), age at diagnosis, sex, Dukes' stage and tumour site were obtained from the cancer registry component of the NCDR. Postal code at diagnosis determined an individual's area-based measure of socioeconomic background using the income domain of the 2004 Index of Multiple Deprivation (IMD).^21^

A primary surgical procedure was sought for every individual using the Hospital Episode Statistics (HES) dataset within the NCDR. HES data do not fully capture information on those treated in non-NHS hospitals (fewer than 8% of the population in England). Procedures were categorised as: major resection; minor resection; bypass; stoma formation; stent insertion (using the Office of Population Censuses and Surveys Classification of Interventions and Procedures V.4)^22^ within 12 months of the diagnosis. If no procedure could be identified, patients were allocated to a ‘no surgical procedure’ group and the attendance to a hospital with a CRC MDT closest to the date of diagnosis (and within 30 days of diagnosis) was taken as their ‘index admission’. Elective or emergency presentation and screening status were identified.^23^ ^24^

All 150 NHS Trusts (a single hospital or group of jointly administered hospitals) with CRC MDTs were included in this study. The annual Trust CRC workload was categorised as low (≤150 cases), medium (151–250) or high (>250). The Trusts which conduct the majority of biomedical and translational cancer research in England are designated as Experimental Cancer Medicine Centres (ECMCs)^25^ and these were flagged (n=18).

Since 2001, the NCRN recorded patients in each trial in its portfolio by hospital Trust. The portfolio includes all later phase randomised clinical trials and other well designed, peer reviewed, studies funded by the UK Government and partners, including, since 2005, certain commercially funded studies.^26^ The colorectal and anal cancer studies included in the portfolio are listed in online supplementary table S1. Trust research participation was described as a ratio or ‘rate’ calculated by dividing the number of patients with CRC entering research studies (NCRN data) by the number of new patients with CRC managed (NCDR data) in each calendar year. The rate was calculated for each Trust both for interventional (defined by the NCRN on portfolio entry as studies which might result in a change in patient management) and observational studies. These rates were applied as the ‘research participation’ variable to all individual patients treated in that Trust in each of the years.

10.1136/gutjnl-2015-311308.supp1 Supplementary data

The relationship between research participation rates and patient with CRC outcomes was explored in two ways. First, the effect of research participation (as described above) on survival was examined using a Cox model, with follow-up time censored at death, 5 years or 30 June 2010 (median follow-up time: 5 years, range: 1.5–5 years). Research participation rates were categorised as 0%, >0%–5%, >5%–10% or >10% and adjusted for age group, sex, IMD quintile, Dukes' stage, tumour site, primary procedure, admission method, screening status, year of diagnosis, annual Trust workload and ECMC status. A second, more complex analysis was undertaken in an attempt to incorporate the two concepts of level of research participation as well as how long it was sustained (see online supplementary files: statistical methods).^27^ ^28^ Each possible rate of participation in interventional clinical research (between 0% and 50%) was used as a cut-off point and for each percentage (‘cut-off’) the number of individual years that each Trust achieved that cut-off was calculated (this did not have be continuous). That gave the required composite score of the percentage cut-off and how well it was sustained, and this was then entered into the Cox model.

In addition to survival analyses, 30-day postoperative mortality was calculated in 142 663 patients who underwent a major resection. Logistic regression was performed to investigate the relationship between postoperative mortality and research participation (using the two methods detailed above) with adjustment as per the survival analyses (with the exception of primary procedure). Missing data for Dukes' stage (23.3%) and IMD (1.5%) were imputed using the ‘ice’ command within Stata with 10 iterations (for both the survival and postoperative mortality analyses). The imputation model included all variables used in the analysis, all variables predictive of missing values and all variables influencing the process causing the missing data.^29^

## Results

**Figure 1:**
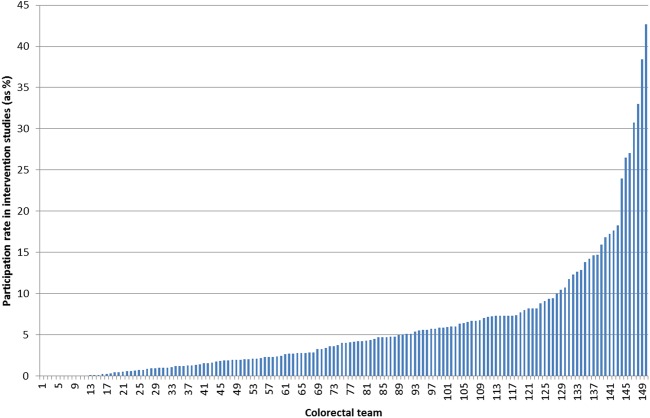
Trust average research participation rates (the numbers of patients enrolled in interventional colorectal cancer (CRC) trials divided by total number of new patients with CRC) over the whole 8-year period expressed as percentages by Trust.

Between 2001 and 2008, 209 968 individuals were diagnosed with CRC in England; their overall 5-year survival was 41.5%. Of these, 142 663 individuals underwent major resection, of whom 6.3% died within 30 days. The characteristics of the population are shown in table 1. Over the same period, 30 998 individuals (14.8%) participated in any NCRN colorectal study and 11 758 (5.6%) participated in an interventional trial. Figure 1 shows the average rate of participation in interventional trials by Trust (patients enrolled/number of new CRC patients, %) over the whole 8-year period; however, this masks the year-to-year variation in participation rates and our subsequent analyses have considered participation rates in each year of the study.

**Table 1:** Characteristics of the study population

	All cases		Major resections	
Variable	n=209 968		n=142 663	
Age group (years)				
<60	38 681	18.4%	28 661	20.1%
60–70	52 643	25.1%	39 271	27.5%
70–80	70 659	33.7%	49 426	34.6%
>80	47 985	22.9%	25 305	17.7%
Sex				
Male	116 050	55.3%	79 276	55.6%
Female	93 918	44.7%	63 387	44.4%
Deprivation quintile				
1 (least deprived)	41 557	19.8%	29 059	20.4%
2	45 121	21.5%	31 302	21.9%
3	44 478	21.2%	30 330	21.3%
4	41 076	19.6%	27 390	19.2%
5 (most deprived)	34 488	16.4%	22 529	15.8%
Missing	3248	1.5%	2053	1.4%
Dukes' stage				
A	20 390	9.7%	16 967	11.9%
B	53 443	25.5%	49 822	34.9%
C	53 778	25.6%	49 150	34.5%
D	33 654	16.0%	12 653	8.9%
Missing	48 703	23.2%	14 071	9.9%
Tumour site				
Colon	148 722	70.8%	104 681	73.4%
Rectum	61 246	29.2%	37 982	26.6%
Primary procedure				
Major resection	142 663	67.9%	142 663	100.0%
Local excision	7399	3.5%		
Bypass	968	0.5%		
Stoma	7899	3.8%		
Stent	2025	1.0%		
No surgical procedure	49 014	23.3%		
Admission method				
Elective	144 645	68.9%	109 344	76.6%
Emergency	65 323	31.1%	33 319	23.4%
Screening status				
Symptomatic	207 941	99.0%	140 965	98.8%
Screen detected	2027	1.0%	1698	1.2%
Year				
2001	18 735	8.9%	12 949	9.1%
2002	25 397	12.1%	17 383	12.2%
2003	25 880	12.3%	17 587	12.3%
2004	26 799	12.8%	18 068	12.7%
2005	27 340	13.0%	18 542	13.0%
2006	28 011	13.3%	18 841	13.2%
2007	28 493	13.6%	19 335	13.6%
2008	29 313	14.0%	19 958	14.0%
Annual Trust workload*				
Low	66 320	31.6%	44 928	31.5%
Medium	74 579	35.5%	50 717	35.6%
High	69 069	32.9%	47 018	33.0%
Trust ECMC status†				
No	182 599	87.0%	124 834	87.5%
Yes	27 369	13.0%	17 829	12.5%

*Annual Trust workload (number of patients with CRC managed) was categorised as: low (≤150), medium (151–250), high (>250).

†Trust ECMC status was categorised as yes or no according to the list of centres provided on the ECMC website (http://www.ecmcnetwork.org.uk/network-centres).

CRC, colorectal cancer; ECMC, Experimental Cancer Medicine Centre.

The rates of participation in interventional studies by year within each Trust (categorised as 0%, >0%–5%, >5%–10%, >10%) showed a significant positive association with improved survival and reduced postoperative mortality (summarised in table 2 and with full results in online supplementary table S2). Patients treated in a Trust with >10% of patients in intervention trials had improved 5-year survival (HR 0.97, 95% CI 0.95 to 0.99 compared with 0% participation) and reduced 30-day postoperative mortality (OR 0.89, 95% CI 0.82 to 0.96 compared with 0% participation).

**Table 2:** Multivariable analysis of the association between intervention trials research participation and 5-year survival and 30-day postoperative mortality using simple categories

Research participation	5-year survival*			30-day mortality†		
	n	HR	95% CI	n	OR	95% CI
None (0%)	63 796	1.00		43 168	1.00	
Low (>0%–5%)	66 829	1.00	0.98 to 1.01	46 002	0.93	0.87 to 0.98
Medium (>5%–10%)	42 932	1.01	0.99 to 1.02	29 185	0.94	0.88 to 1.00
High (>10%)	36 411	0.97	0.95 to 0.99	24 308	0.89	0.82 to 0.96

*Based on 209 968 patients; adjusted for age group, sex, deprivation quintile, Dukes’ stage, tumour site, primary procedure, admission method, screening status, year of diagnosis, annual Trust workload, ECMC status. For the full model results see online supplementary table S2.

†Based on 142 663 patients; adjusted for age group, sex, deprivation quintile, Dukes’ stage, tumour site, admission method, screening status, year of diagnosis, annual Trust workload, ECMC status. For the full model results see online supplementary table S2.

ECMC, Experimental Cancer Medicine Centre.

**Figure 2:**
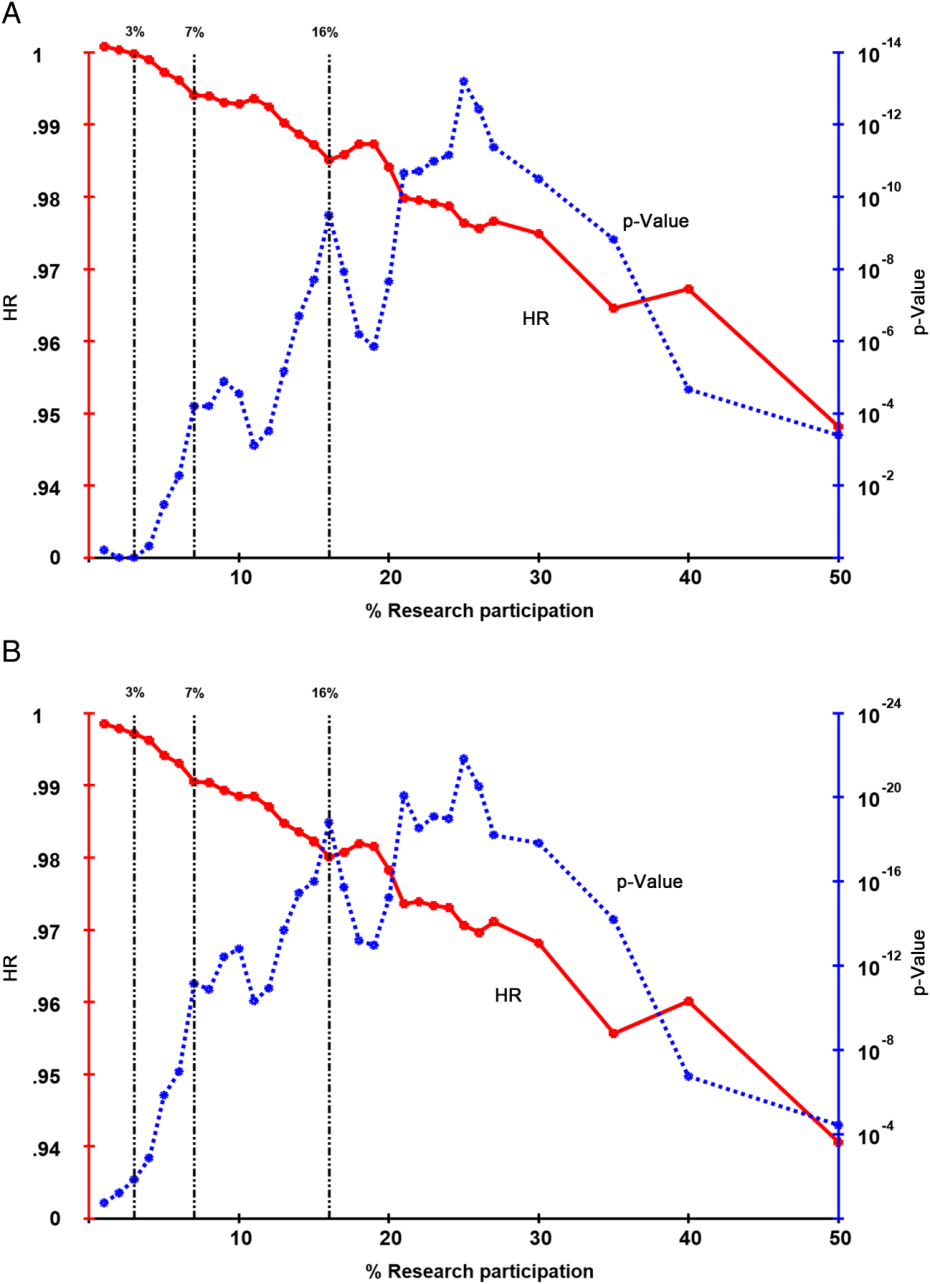
HR and p value plots showing the effect of an increasing sustained rate of Trust-level research participation in CRC studies on 5-year survival. Cox multivariable analysis was performed using the explanatory variables listed in the text. The additional variable was a composite score derived from the number of years for which the research participation rate met and exceeded the % cut-off, giving the number of years the rate of participation was sustained above the percentage shown. The HR shown is for each year where the rate was sustained above that percentage. The associated p value is also shown, plotted on a log scale. (A) Includes adjustment for Experimental Cancer Medicine Centre (ECMC) status while (B) excludes adjustment for ECMC status. Where 3% of patients participate in clinical trials there is a significant (p<0.01) impact on 5-year survival. There is a rapid increase in the p value as the percentage research participation increases up to 7% (p<10^11^) and then a slower increase to a peak or peaks between 16% and about 30%. After this the p value decreases, as the number of Trusts achieving such high levels of research participation becomes smaller. The same pattern is seen for both analyses (with and without ECMC status).

Figure 2 shows the results of the second, more complex, analysis using the full range of ‘cut-offs’ and incorporating the number of years a Trust achieved that cut-off. The HRs and associated p value for each cut-off (0%–50%) and the number of years that each Trust achieved that cut-off, were derived from the Cox model. The HRs decrease with increasing cut-off points used in the Cox model. While the cut-off with the largest impact on survival was 25% participation, a second peak occurred at 16% (figure 2A). As research participation increases, the proportion of patients to which this applies reduces. It is of most relevance to maximise this proportion of patients and look at the lowest sensible threshold of research participation which still results in a model fit that is close to the optimum. As such, 16% was used to define ‘high participation’. This analysis was repeated, without the inclusion of ECMC status (figure 2B). The impact of clinical research participation was still highly significant and followed the same pattern, with similar peaks in the p value.

Using the 16% threshold, no Trusts achieved this level of participation in 2001. Between 2002 and 2008, 41 Trusts recruited at this level for one or more years, with high research interventional participation being greatest in 2003 (27 Trusts). Of the 18 ECMCs, 11 achieved the high participation rate in at least 1 year. However, most of the Trusts that achieved this high participation threshold were not ECMCs (table 3). The breakdown of the institutions achieving 3%, 7% or 16% participation by the number of years above each threshold is given in table 3. The 16% level of participation is only achieved by a minority of Trusts and is difficult to sustain. However, 7% is achieved by most Trusts and 3% by almost all.

**Table 3:** Proportion of patients achieving 3%, 7% or 16% participation and the number of years above each threshold

Sum of years above participation threshold										
	0	1	2	3	4	5	6	7	8	Total
% participation in NHS Trust										
≥3%	12.0	9.9	14.5	9.0	9.2	8.1	15.5	20.0	1.7	100
≥7%	33.1	17.1	11.2	13.6	9.9	6.4	3.3	5.5	0.0	100
≥16%*	71.9	12.0	7.0	3.5	3.1	0.8	0.3	1.4	0.0	100

*For the three selected cut-off points, the percentage of all patients who were managed in a Trust which achieved that cut-off for between 0 and 8 years is shown. A total of 41 out of 150 Trusts achieved high (≥16%) participation for one or more years; 11 of the 18 ECMCs achieved this rate of participation.

ECMC, Experimental Cancer Medicine Centre.

**Figure 3:**
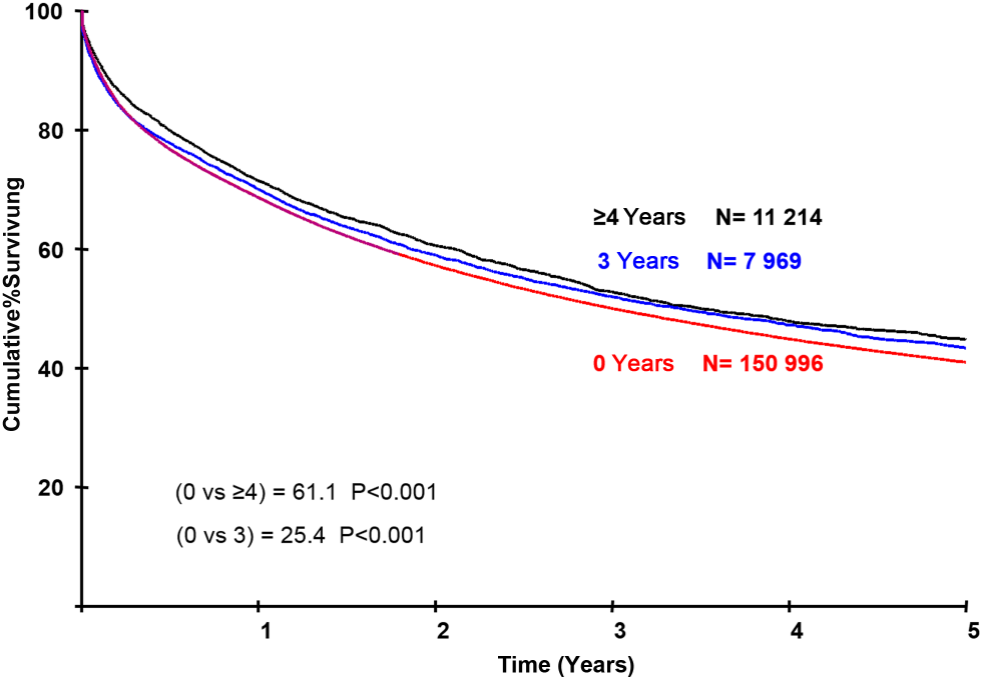
Adjusted survival curves for patients treated in institutions with high research participation. It shows the cumulative survival for patients treated in institutions that have ≥16% participation in interventional clinical trials for 0, 3 or ≥4 years. At the scale of this graph the results for 1 and 2 years are superimposable over that for 0 years. The curves are highly significantly different and show that the separation occurs principally in the first year of follow-up. Survival is adjusted for primary procedure, index admission, Dukes’ stage, age, deprivation and Experimental Cancer Medicine Centre status.

Multivariable analysis showed that treatment in a Trust with high interventional research participation (≥16% in any individual year) was associated with an improvement in 5-year survival (adjusted HR=0.95, 95% CI 0.92 to 0.97) (summarised in table 4 and full results in online supplementary table S3) for all patients. Survival increased with the number of years a Trust had high rates of participation (adjusted HR=0.90, 95% CI 0.88 to 0.93 ≥4 years compared with 0 years) (table 4 and figure 3). This represents a 3.8% absolute difference in survival (41.0% and 44.8% in the institutions with 0 and ≥4 years high research participation, respectively) as can be seen graphically (figure 3). The main improvement occurred over the first 6–8 months after diagnosis, reflecting the early management of CRC including the reduction in postoperative mortality. All analyses were adjusted for year of diagnosis. The impact on survival of being treated in a Trust with high (≥16%) interventional trial participation was separately significant for patients diagnosed in each of the years 2004, 2006, 2007 and 2008 (see online supplementary table S4).

**Table 4:** Multivariable analysis of the association between intervention trials research participation and 5-year survival and 30-day postoperative mortality using an optimal cut-point approach

	5-year survival*			30-day mortality†		
	n	HR	95% CI	n	OR	95% CI
	*Participation threshold (*≥*16% in any individual year)*					
Low (<16%)	192 755	1.00		131 364	1.00	
High (≥16%)	17 213	0.95	0.92 to 0.97	11 299	0.85	0.78 to 0.94
	*Number of years with high participation*					
0 years	150 996	1.00		102 321	1.00	
1 year	25 110	0.99	0.97 to 1.00	17 769	0.95	0.89 to 1.02
2 years	14 679	1.01	0.98 to 1.03	10 360	0.93	0.85 to 1.02
3 years	7407	0.90	0.87 to 0.93	4879	0.87	0.76 to 0.99
≥4 years	11 776	0.90	0.88 to 0.93	7334	0.76	0.67 to 0.86

*Based on 209 968 patients; adjusted for age group, sex, deprivation quintile, Dukes’ stage, tumour site, primary procedure, admission method, screening status, year of diagnosis, annual Trust workload, ECMC status. For the full model results see online supplementary table S3.

†Based on 142 663 patients; adjusted for age group, sex, deprivation quintile, Dukes’ stage, tumour site, admission method, screening status, year of diagnosis, annual Trust workload, ECMC status. For the full model results see online supplementary table S5.

ECMC, Experimental Cancer Medicine Centre.

Trusts with high rates of interventional research participation (≥16% in any individual year) were also associated with a reduction in the adjusted odds of death within 30 days of surgery (adjusted OR=0.85, 95% CI 0.78 to 0.94) (summarised in table 4 and with full results in online supplementary table S5). The odds of postoperative death decreased as the number of years with high research participation increased, with individuals treated in Trusts with ≥4 years high participation having the lowest mortality (OR=0.76, 95% CI 0.67 to 0.86 compared with 0 years). This represents an absolute difference of 1.5% in 30-day postoperative mortality (from 6.5% to 5.0% in the Trusts with 0 years and ≥4 years, respectively).

For both 5-year survival and 30-day postoperative mortality, a sensitivity analysis was performed including only patients with no missing data, a ‘complete case analysis’. This was compared with the analysis with missing data imputed and there were no substantial differences (see online supplementary table S6). The impact of interventional research participation on 1-year survival using the 16% cut-off was highly significant (see online supplementary table S7).

It was not possible in the whole dataset to test the impact of research participation upon processes of care other than surgery. A regional subset of the NCDR data (30 701 patients), for which chemotherapy data were available, showed increased uptake of chemotherapy in Trusts with higher interventional research participation (≥16%) compared with those with low participation (<16%) (OR 1.13, 95% CI 1.00 to 1.27).

High participation rates in ‘observational’ studies were not associated with improved survival or postoperative mortality (results not shown). Closer inspection of the data revealed that most of the patients in the observational studies were recruited several years after their original diagnosis, into genetics studies. These data were not, therefore, studied further.

## Discussion

This large population-based study in a big national unselected dataset, using CRC as an example, supports the prior hypothesis that being treated in Trusts with sustained high participation in interventional clinical research is independently associated with better outcomes. This effect is seen across all NHS Trusts that care for patients with CRC and is not restricted to academic centres or large institutions. Using an arbitrary cut-point of >10% new cases to define high research participation, there was an improvement in 5-year survival for patients treated in high participation Trusts compared with those with no research activity. Using a more sophisticated approach, higher rates of participation and more years (of the eight studied) with high participation each showed a ‘dose effect’ with, for example, an estimated 3.8% improvement in survival for patients treated in Trusts achieving ≥16% research participation for 4 years or more. However, lower thresholds—for example, 7% participation—still produce highly significant benefits over no research participation.

How does the impact of sustained high research participation compare to other interventions? The maximum size of the observed impact of research participation on survival is comparable to the whole patient population impact seen following a highly positive intervention trial, where increments in survival rarely exceed 5%, and the population impact is usually less than the increment seen in the trial. Alternatively, the addition of adjuvant chemotherapy to the treatment of patients with Duke's stage C CRC results broadly in a 10% increase in their long-term survival—one of the substantial advances in the management of this disease in recent decades. Duke's C cases are some 30% of all cases. The benefits of this adjuvant chemotherapy for the whole CRC population are thus comparable to the potential benefits of high research participation.

Crucially, the effect of research activity is seen after adjustment for medical and social factors such as casemix, hospital case volume and ECMC status, which may be expected to affect the performance of different institutions.^30^ Similarly, the effect is independent of year of diagnosis, not simply a reflection of the general improvement in CRC outcomes over time. The increase in research participation precedes the onset of the increase in survival with which it is independently associated. This pattern is what would be expected if there were a causal link.

It might be assumed that the highly research-active institutions are limited to the large ‘centres of excellence’ but this is not the case. Centres of excellence do perform well; 11 out of 18 ECMCs achieve high participation. However, their contribution is not the sole component of the impact on the whole NHS and 30 high participation Trusts are not ECMCs.

The lower 30-day mortality in research-active Trusts could reflect better diagnosis, staging or surgical and perioperative care; the sustained improvement at 5 years reflects all aspects of care. The impact of the novel treatments among trial participants cannot account for the observed effect on overall survival: of the 35 intervention studies open during our study only six produced significant positive effects on survival, and even in the most research-intensive Trusts the novel arms of those trials account for only a small proportion of the total CRC population.

Centres that are active in research are more likely to have broader diagnostic and therapeutic arsenals. The development of this ‘arsenal’ could be a preceding condition that led to greater trials participation. On the other hand, greater trials participation might lead through to a stronger diagnostic and therapeutic arsenal which would be one component of the mechanisms by which the trials resulted in improved outcomes. Association studies alone cannot fully resolve this question.

This study has several limitations. Studies were categorised as interventional or observational by NCRN, who classified some studies of prevention and of follow-up regimens, as ‘interventional’. We believe it is preferable to use the NCRN classification rather than develop our own, but a narrower definition of interventional may have given higher levels of significance. Anal cancer trials were included in the NCRN CRC list as patients with anal cancer are managed by the same MDTs who treat CRC. The number of patients with anal cancer included in trials is very small and exclusion of these is unlikely to have any impact on the results.

Patients were allocated to Trusts according to where they received their primary procedure. In the UK, patients rarely travel far for primary treatment and confounding based on self-referral will be minimal. Important care process data, such as use of chemotherapy, was only available for a subset of the whole sample. Full 5-year follow-up was not available for all patients due to the time-lag associated with obtaining complete cancer registration data, but most deaths occur within the early follow-up period so any effect would be minimal. Analyses of 1-year survival with 100% follow-up showed similar results (see online supplementary table S7).

Only 12 Trusts achieved an average of ≥16% recruitment to trials across the study period and it could be argued that these results are based on a limited subgroup of Trusts. However, the analysis looked at recruitment within each year separately and, as a result, a much higher number of Trusts were categorised as having high research participation: 41 Trusts recruited ≥16% of patients for one or more years. While this analysis identified 16% as an ‘optimum’ recruitment figure, in reality this may be difficult to achieve and smaller increases in participation are still associated with significant improvements in survival. Table 3 and figure 2 demonstrate that Trusts with participation rates of 3% or 7% also show highly significant associations with improved outcomes.

Although we have shown an association between research participation and survival in a very large unselected dataset, we must be cautious when we seek to infer a causal contribution. However, a randomised trial of ‘research versus no research’ is not possible. This natural experiment, presented by the rapid expansion of trial activity across a whole national health system, is perhaps the best opportunity to address the subject through outcomes research.^31^ It is reassuring that the association of research participation with survival is independent of casemix, case volumes and ECMC status, but we must acknowledge the possibility of residual confounders. Our prior hypothesis and analysis plan concerned the impact of interventional clinical research, and we were unable to examine the impact of observational clinical studies in this dataset. Finally, CRC results in England, although improving steadily, are less good than comparable countries,^32^ ^33^ which may affect the applicability of our findings in countries with the best outcomes.

Our results allow investigators to show patients, healthcare commissioners and policymakers that being treated in a hospital active in clinical research is strongly associated with better outcomes. They provide an indication that increasing clinical research may be an important tool for improving hospital performance. The data support this general principle. They do not indicate that all trials should be conducted across the whole NHS—the best locations for a trial will be dependent on the technologies involved and the capacity and capability of each Trust. When considered alongside other studies and reviews which suggest research participation improves processes of care,^6–12^ ^34^ our data provide an added incentive to integrate research into standard medical care. The association between research participation and outcomes is strong, grows steadily with increasing and sustained participation, and that onset of the improvements in outcomes follow the onset of increased participation in a timely, plausible manner. However, this observation needs to be reproduced in other datasets and diseases. Future research will test the generalisability and specificity of our findings on other cancers and non-malignant diseases, and will also study in more depth the nature of the relationship between research participation and outcomes.
