# Supplementary material for: High hospital research participation and improved colorectal cancer survival outcomes: a population-based study
Source: Gut. 2016 Oct 20;66(1):89–96. doi: 10.1136/gutjnl-2015-311308 (PMC5256392; doi:10.1136/gutjnl-2015-311308)
Supplement: Supplementary data [file gutjnl-2015-311308supp.pdf]

SUPPLEMENTARY FILES PROPOSED FOR ON-LINE PUBLICATION

Statistical Methods

Cox regression was used to investigate the relationship between five-year survival in individual hospital Trusts and research participation rates of those Trusts, using the methods of classifying research participation described in the text. Additional explanatory variables included in the regression analysis were age at diagnosis (<60 years, 60-70 years, 70-80 years, 80+ years), sex, IMD income quintile, Dukes' stage, tumour site (colon, rectum), primary procedure as described in the text, method of presentation (elective, emergency), screening status (screen-detected, symptomatic), year of diagnosis (2001-2008), annual Trust workload (low, medium, high) and ECMC status (yes/no). Adjusted survival curves,[27] which do not assume proportional hazards and therefore can show time-related effects clearly, were used and differences between curves were plotted, adjusted for factors found significant in the Cox regression.

Multi-level models were investigated to account for the hierarchical nature of the data (patients nested within Trusts), but it was not possible to extend the multi-level approach to the imputed data and the survival analysis due to the volume of data (over 2 million records in the imputed dataset) and the computational power required. Single-level models with imputation were chosen as this allowed the same methodology to be used for both the survival and post-operative mortality analyses.

Our prior hypothesis was that the relationship between interventional clinical research participation and outcomes would be dependent upon both the degree and duration of research participation, with best results coming from institutions (trusts/hospitals) which had high levels of research participation sustained over a number of years. These two variables are inextricably linked, since duration can only be calculated once a degree (percentage) of

research participation has been chosen. The second “complex” multivariable analysis examined the effect of participation rates above a wide range of thresholds and the duration (years) of participation rates above each threshold. Sustained research participation was assessed by calculating the number of years (of the eight studied) during which the institutions’ recruitment rates were sustained above the particular threshold cut-point. This enabled the identification of an optimum threshold for use in the analysis.

To evaluate this composite relationship therefore, while adjusting for the other explanatory variables such as age, stage, primary procedure etc., it was necessary to consider a range of participation percentages which would constitute a ‘high’ level of research, and then count the number of years (of the 8 studied) during which the institutions’ recruitment was sustained above that percentage (main paper Figure 2).

Formally, calculations proceeded as follows:

a) a percentage cut-off was chosen to represent high research activity (ranging from 1% to 50%)

b) the number of years (out of the 8 studied) for which research participation was greater than this percentage was counted for each institution, and therefore for each patient, generating a new variable for each patient, with values ranging from 0 to 8

c) it was noted that this variable therefore represents the required composite since it combines duration of research participation with degree of research participation above a particular percentage

d) this new composite ‘sum of years’ variable was included as an additional explanatory variable in a Cox model of survival which includes all the other variables (listed for example in Table 1)

e) the multivariate chi-square for this new variable was evaluated from this Cox model with all the other variables being included - this therefore represents the significance of research participation sustained above this threshold

f) the Cox model chi-squares were used to generate the p values and were plotted, to evaluate this composite relationship. This plot of the p values derived from this analysis are shown in the main paper, Figure 2.

Note that if there is a relationship between research activity and outcomes it could have a variety of forms. It could be simply that the higher the research activity the better the outcomes (perhaps with a simple linear relationship, or more likely a more complicated relationship such as a Gompertzian pattern); it could be that research activity has to rise above some threshold in order for outcomes to improve, but that there is no further improvement once this threshold is achieved; or there could be a threshold with further improvements as research participation increases beyond this threshold. These possible patterns could well apply to both the level of research participation and its duration. The plot shown in Figure 2 evaluates these potential patterns, with adjustment for all the other relevant variables. The gradual increase in significance as the threshold is raised implies that the first of these patterns is likely to hold, with the higher the research activity the better the outcomes. The 'sum of years' variable was included in the Cox model in a simple linear fashion, although other relationships were considered. The numbers of patients having >4 years of research participation were limited, especially above the higher research participation percentages, making it difficult to evaluate more sophisticated models for this relationship.

Following on from this observation, the resulting choice of optimum threshold for use in the main analysis was derived from finding an appropriate balance between the maximum at

about 25%, and a percentage with a similarly high Cox model chi-square which delineated the largest possible proportion of the population. This was observed to occur at 16%.

Cut-point approach methods

Previously developed methods assessed and quantified the, relatively minor, effects on the type I error (reflected in the Cox model χ^2) of this cut-point optimisation. A Simulation approach, as described in more detail in Viprey et al,[28] demonstrated that, in this particular case with 210,000 patients, there was a penalty amounting to a reduction of approximately 5 in the χ^2 Cox model statistic for having optimised the cut-point when examining the effect of research activity on survival. Therefore a χ^2 of 80 (see for example Supplementary Figure 1) should be reduced to approximately 75 to reflect the fact that an optimum cut-point was derived. Given the large dataset involved and the magnitude of the effects observed, the use of this optimum cut-point therefore makes little or no material difference to the conclusions drawn, or to the reported effect sizes. Note that the other way in which the optimum cut-point approach could over-inflate the magnitude and significance of the result is if there were anomalously large 'spikes' in the significance levels for particular cut-points, though this is taken account of, to a large degree, in the simulation approach. Anyway, by showing the results for the full range of cut-points used, any such anomalous spikes can be seen and taken into account.

To elaborate on the simulation method, Supplementary Figure 1 displays the distribution of χ^2 statistics for the case when there is no effect of research activity (i.e. the null hypothesis) - considering two cases; when the research activity variable is examined to see if it might have a continuous relationship to survival, and then for the equivalent threshold effect model on survival using an optimal cut-point (run with 100 cut-points for each simulation). 10,000 simulations were run with 210,000 patients in each. The shape of this null-hypothesis cut-

point histogram reflects the fact that when there is no effect it is more likely that there exists a cut-point that has some effect compared to there being an effect in the continuous model. There is an increase of 5 in the χ^2 values comparing a 95% range for the two models (-3.9 & +3.9 for the continuous model, -8.8 & +8.8 for the cut-point model). Similarly, the 99% range is 5 larger for the cut-point model, leading to the conclusion that the χ^2 should be reduced by about 5 as a penalty for examining 100 cut-points and choosing the optimum.

Note that if there was a continuous effect, with this large a number of patients, then the continuous model would always fare considerably better than the cut-point model, because the true nature of the continuous effect would be lost by using a cut-point; optimising the cut-point only compensates for this to a very minor degree. If the true nature of the effect really was a threshold/cut-point effect, then of course the cut-point model would be better (and appropriate), as observed in this particular case. So the argument that the cut-point approach randomly inflates the type I error by looking at all cut-points becomes less and less valid, as the dataset becomes larger, unless there is a very small effect size. The only remaining precaution necessary to avoid over-interpretation of the cut-point results with such a large dataset is to ensure that the chosen cut-point does not occur at a particular unusually high ‘spike’, and Figure 1 shows this is not the case.

Supplementary Tables

Supplementary Table 1: Details of NCRN portfolio colorectal cancer studies recruiting between 2001 and 2008

Supplementary Table 2: Multivariable analysis of the association between intervention trials research participation and five-year survival and 30-day post-operative mortality using simple categories – Full results

Supplementary Table 3: Multivariable analysis of the association between intervention trials research participation and five-year survival using an optimal cut-point approach – Full results of the threshold and duration analyses

Supplementrary Table 4: Association between high intervention trials research participation for each separate calendar year and five-year survival

Supplementary Table 5: Multivariable analysis of the association between intervention trials research participation and 30-day post-operative mortality using a model-derived cut-point – Full results of the threshold and duration analyses

Supplementrary Table 6: Comparison of the complete case and imputed multivariable analyses of five-year survival and 30-day post-operative mortality (low vs. high research participation)

Supplementary Table 7: Multivariable analysis of the association between intervention trials research participation and one-year survival using an optimal cut-point approach

Supplementary Table 1: Details of NCRN portfolio colorectal cancer studies recruiting between 2001 and 2008

Study	Type	Total no. patients	No. in present analysis	Primary endpoint(s)/ aims(s)	Details/results
**ACT II** - Chemoradiation and maintenance therapy for patients with anal cancer	Interventional	940	917	Complete response<br>3-year progression-free survival	No significant difference between treatment arms.
**Big ET Study** - Endothelin levels in patients with Colorectal Cancer	Interventional	77	17	Endothelin levels	No prognostic value.
**CAPP2 Study** - Colorectal polyp and cancer prevention using aspirin and resistant starch in carriers of HNPCC (Lynch Syndrome)	Interventional	861	68	Colorectal cancer incidence	Non-significant reduction in incidence.
**CAPP-IT** - The role of pyridoxine in controlling capecitabine induced hand-foot syndrome	Interventional	106	104	Dose modification of capecitabine at 12 weeks	Reduction in hand-foot syndrome and the need for lower dosage of chemotherapy.
**CHRONICLE** - Chemotherapy or no chemotherapy after neoadjuvant treatment in locally advanced rectal cancer	Interventional	113	97	Disease-free survival	Not yet reported.
**CLASICC** - Conventional versus Laparoscopic-Assisted Surgery in Colorectal Cancer	Interventional	794	179	Margins<br>% Dukes C2<br>In-hospital mortality	No difference in outcomes.
**CLOCC** - Local treatment of liver metastases by radiofrequency combined with chemotherapy versus chemotherapy alone	Interventional	119	6	30-month survival	No difference in survival.
**COIN** - Comparing either COntinuous chemotherapy plus cetuximab or INtermittent chemotherapy with standard therapy	Interventional	2,445	1,959	Overall survival<br>Non-inferiority overall survival	+/- cetuximab - no difference.<br>Continuous vs. intermittent treatment – non-inferiority not met.
**COIN QoL Sub-Study** - Quality of Life Sub-Study	Observational	Unknown	20		No information located.
**COIN-B / CR11** - intermittent chemotherapy plus continuous or intermittent cetuximab in patients with metastatic colorectal cancer	Interventional	169	105	Incorporation of cetuximab	Cetuximab was safely incorporated in the two treatment strategies. Results require validation in phase III trials.
**CR07** - Pre-operative radiotherapy and selective post-operative chemoradiotherapy in rectal cancer	Interventional	1350	653	Local recurrence	***Significant reduction in local recurrence.**
**Deferral of Surgery** - Timing and deferral of rectal surgery following a continued response to pre-operative chemoradiotherapy	Observational	On-going	8	2-year failure rate	Still recruiting.

**Enhanced Recovery Trial** - Multi-modal care pathway for patients undergoing surgical resection for colorectal cancer	Interventional	60	58	Length of hospital stay<br>Complications<br>Readmissions	Enhanced recovery package associated with reduced hospital stay with no adverse outcomes.
**EnROL** - Conventional versus laparoscopic surgery for colorectal cancer within an Enhanced Recovery Programme	Interventional	204	12	Post-operative fatigue	Not yet reported.
**EORTC QLQ-CR29** - An international study to test the EORTC QLQ-CR29 in patients with colorectal cancer	Observational	351	70	Testing of questionnaire	Valid and reliable.
**EORTC/GITCCG 40983** - Pre and post-operative chemotherapy with oxaliplatin, 5FU/LV versus surgery alone in resectable liver metastases	Interventional	364	63	Progression-free survival	***Non-significant intention-to-treat analysis. Improvement in survival for eligible and resected patients.**
**EXPERT-C** - Oxaliplatin, capecitabine and pre-operative radiotherapy with or without cetuximab followed by total mesorectal excision in high risk rectal cancer	Interventional	165	78	Complete response	***Primary end point not met but survival difference shown.**
**EXTRA** - Evaluation of Xeloda Treatment with radiotherapy in Anal Cancer	Interventional	31	18	Local control at 6 months	***End point met. Acceptable toxicity and efficacy.**
**FAB2** - The impact of folate and its interaction with riboflavin on biomarkers in colorectal cancer risk	Interventional	204	47	Measurement of biomarkers	Evidence of biomarker response but no difference between the healthy and polyp groups.
**FACS** - The cost-effectiveness of intensive versus no scheduled follow-up in patients who have undergone resection for colorectal cancer	Interventional	1,202	1,077	Surgical treatment of recurrence	***Significant for the 3 more intensive arms vs. minimal follow-up. Factorial comparison – no difference.**
**FOCUS** - The role of irinotecan and oxaliplatin in advanced colorectal cancer	Interventional	2,135	1,387	Overall survival<br>Non-inferiority overall survival	Starting treatment with a single drug limits toxicity without compromising benefit.
**FOCUS2** - Drug treatment for bowel cancer: making the best choices when a milder treatment is needed.	Interventional	459	409	Progression-free survival<br>Global quality of life	Milder treatments are comparable.
**FOxTROT** - Fluoropyrimidine, Oxaliplatin & Targeted Receptor pre-Operative Therapy for in high-risk operable colon cancer.	Interventional	On-going	14	Recurrence at 2 years	Still recruiting.
**Genetic Factors in Colorectal Cancer** - The role of genetic factors in clinical outcome for colorectal cancer patients	Observational	Unknown	309	Genes associated with survival	Not yet reported.

**MERCURY** - Magnetic Resonance Imaging and rectal cancer European equivalence study	Observational	679	387	Equivalence in extramural spread	No difference.
**MERCURY 2**- Low Rectal Cancer Study	Observational	On-going	32	Margins	Still recruiting.
**Molecular pathology of colorectal cancer** - The role of microRNA's and their molecular targets in colorectal cancer progression	Observational	On-going	5	Response to treatment and molecular factors.	Still recruiting.
**New EPOC** – pre- and post-operative treatment of resectable colorectal liver metastases requiring chemotherapy	Interventional	272	30	Progression-free survival	Inferior for experimental arm. Stopped early for futility.
**NSCCG** - National Study of Colorectal Cancer Genetics	Observational	On-going	12,951	Genes associated with development of cancer	Still recruiting.
**ORBIT** - Effective management of radiation-induced bowel injury: A randomised controlled trial	Interventional	218	53	Quality of life	Targeted intervention resulted in improvement in symptoms vs. usual care.
**OxaliCap**-RT - Integrating intravenous oxaliplatin plus oral capecitabine with pelvic radiation for rectal cancer	Interventional	19	16	Dose per fraction of RT<br>Compliance	Closed early.
**PACT** - Patient Preferences in Adjuvant Colorectal Cancer Therapy	Interventional	40	40	Patient preference	Closed early. Increased acute toxicity.
**PICCOLO** - Treatment for fluorouracil-resistant advanced colorectal cancer	Interventional	1,198	532	Overall survival	No difference between groups.
**QUASAR** - Quick and Simple and Reliable: A Study of Colorectal Cancer Treatment	Interventional	3,239	439	All-cause mortality	***Small survival benefit from adjuvant chemotherapy.**
**QUASAR 2** - Multicentre international study of capecitabine +/- bevacizumab as adjuvant treatment of colorectal cancer.	Interventional	1,892	889	Disease-free survival	Not yet reported.
**RICE (NWCOG - 2)** - A phase I/II study of Radiotherapy, Irinotecan, Capecitabine then Excision for locally advanced rectal cancer	Interventional	Unknown	52	Dose escalation<br>Side-effects	Showed acceptable acute toxicity and morbidity with encouraging response and curative resection rates.
**SCOT** - Short Course Oncology Therapy - A study of adjuvant chemotherapy in colorectal cancer by the CACTUS & QUASAR 3 Groups	Both	600	66	Disease-free survival	Not yet reported
**SIGGAR1** - CT colonography, colonoscopy, or barium enema for diagnosis of colorectal cancer in older symptomatic patients	Observational	5,448	5,403	Diagnosis of colorectal cancer/large polyp<br>Rate of additional colonic investigation	CT colonography more effective at finding cancers/polyps but more unnecessary follow-up tests.
**Sildenafil citrate study** - Efficacy of sildenafil citrate in men with erectile dysfunction after	Interventional	Unknown	8	Improvement in erectile dysfunction	Trial stopped – unable to recruit enough patients.

pelvic surgery for rectal carcinoma					
**The role of biofeedback in improving continence after anterior resection**	Interventional	121	121	Cleveland Clinic Incontinence Score at 1 year	No difference between groups at 1 year.
**Tumour Angiogenesis** - In Non-small cell Lung, Colorectal and Breast Cancer	Observational	On-going	55	Tumour angiogenesis	Still recruiting.
**ukCAP** - Aspirin and / or folate supplementation for the prevention of recurrent colorectal adenomas	Interventional	945	143	Diagnosis of colorectal adenoma	Lower risk of recurrence with aspirin but not folate.
**VICTOR** - Rofecoxib (VIOXX) in colorectal cancer patients following potentially curable therapy.	Interventional	2,464	2,072	Overall survival	Closed early – negative results
**W.O.R.M.S** - Intraoperative fluid volume optimisation using oesophageal Doppler cardiac output measurement	Interventional	128	27	Length of stay<br>Morbidity	Reduction in hospital stay, reduced morbidity
**XERXES** - Early neoadjuvant and synchronous Erbitux in preoperative chemo-radiotherapy using Xeloda followed by excisional surgery	Interventional	Unknown	2	Acute toxicity<br>Compliance	Closed early.

Supplementary Table 2: Multivariable analysis of the association between intervention trials research participation and five-year survival and 30-day post-operative mortality using simple categories – Full results

		Adjusted five-year survival*			Adjusted 30-day mortality*	
**Variable**		**HR**	**95% CI**		**OR**	**95% CI**
Research participation	None (0%)	1.00		None (0%)	1.00	
	Low (>0-5%)	1.00	0.98-1.01	Low (>0-5%)	0.93	0.87-0.98
	Medium (>5-10%)	1.01	0.99-1.02	Medium (>5-10%)	0.94	0.88-1.00
	High (>10%)	0.97	0.95-0.99	High (>10%)	0.89	0.82-0.96
Age group	<60 years	1.00		<60 years	1.00	
	60-70 years	1.31	1.28-1.34	60-70 years	2.27	2.04-2.52
	70-80 years	1.84	1.80-1.87	70-80 years	4.81	4.36-5.31
	>80 years	2.58	2.53-2.63	>80 years	9.83	8.91-10.85
Sex	Male	1.00		Male	1.00	
	Female	0.92	0.91-0.93	Female	0.75	0.71-0.78
Deprivation quintile	1 (least deprived)	1.00		1 (least deprived)	1.00	
	2	1.05	1.03-1.08	2	1.08	1.00-1.16
	3	1.11	1.09-1.13	3	1.12	1.04-1.21
	4	1.15	1.03-1.18	4	1.24	1.15-1.33
	5 (most deprived)	1.21	1.19-1.24	5 (most deprived)	1.39	1.29-1.49
Dukes' stage	A	1.00		A	1.00	
	B	1.54	1.49-1.60	B	1.12	1.02-1.23
	C	2.99	2.90-3.09	C	1.44	1.31-1.58
	D	6.36	6.16-6.58	D	2.04	1.83-2.27
Tumour site	Colon	1.00		Colon	1.00	
	Rectum	0.88	0.87-0.89	Rectum	1.14	1.07-1.21
Primary procedure	Major resection	1.00				
	Local excision	1.68	1.62-1.75			
	Bypass	2.84	2.65-3.03			
	Stoma	2.27	2.21-2.34			

	Stent	1.85	1.76-1.94			
	No surgical procedure	2.25	2.22-2.29			
Admission method	Elective	1.00		Elective	1.00	
	Emergency	0.95	1.93-1.98	Emergency	4.00	3.81-4.20
Screening status	Symptomatic	1.00		Symptomatic	1.00	
	Screen-detected	0.46	0.40-0.53	Screen-detected	0.51	0.37-0.81
Year	2001	1.00		2001	1.00	
	2002	0.97	0.95-1.00	2002	1.02	0.93-1.12
	2003	0.95	0.93-0.98	2003	1.00	0.91-1.11
	2004	0.91	0.89-0.94	2004	1.01	0.91-1.11
	2005	0.91	0.89-0.94	2005	0.89	0.81-0.98
	2006	0.89	0.87-0.91	2006	0.91	0.82-1.00
	2007	0.88	0.86-0.91	2007	0.85	0.77-0.94
	2008	0.86	0.83-0.88	2008	0.80	0.72-0.88
Annual trust workload	Low	1.00		Low	1.00	
	Medium	1.00	0.98-1.02	Medium	0.92	0.87-0.97
	High	1.00	0.99-1.02	High	0.93	0.88-0.99
Trust ECMC status**	No	1.00		No	1.00	
	Yes	0.93	0.91-0.94	Yes	0.86	0.80-0.92

*The models have been adjusted for all factors listed in the table.

**The data were not materially altered whether or not patients managed within the hospital which lost its ECMC status in 2012 were included.

Supplementary Table 3: Multivariable analysis of the association between intervention trials research participation and five-year survival using an optimal cut-point approach – Full results of the threshold and duration analyses

	Participation threshold (≥16% in any year)			Number of years with high participation		
	Adjusted five-year survival*			Adjusted five-year survival*		
Variable		HR	95% CI		HR	95% CI
Research participation	Low (<16%)	1.00		0 years	1.00	
	High (≥16%)	0.95	0.92-0.97	1 year	0.99	0.97-1.00
				2 years	1.01	0.98-1.03
				3 years	0.90	0.87-0.93
				≥4 years	0.90	0.88-0.93
Age group	<60 years	1.00		<60 years	1.00	
	60-70 years	1.31	1.28-1.34	60-70 years	1.31	1.28-1.34
	70-80 years	1.84	1.80-1.87	70-80 years	1.83	1.80-1.87
	>80 years	2.58	2.53-2.63	>80 years	2.58	2.53-2.63
Sex	Male	1.00		Male	1.00	
	Female	0.92	0.91-0.93	Female	0.92	0.91-0.93
Deprivation quintile	1 (least deprived)	1.00		1 (least deprived)	1.00	
	2	1.05	1.03-1.08	2	1.05	1.03-1.08
	3	1.11	1.09-1.13	3	1.11	1.09-1.13
	4	1.15	1.13-1.18	4	1.15	1.13-1.18
	5 (most deprived)	1.21	1.19-1.24	5 (most deprived)	1.21	1.19-1.24
Dukes' stage	A	1.00		A	1.00	
	B	1.54	1.49-1.60	B	1.54	1.49-1.60
	C	3.00	2.90-3.10	C	3.00	2.90-3.10
	D	6.37	6.16-6.58	D	6.37	6.16-6.58
Tumour site	Colon	1.00		Colon	1.00	
	Rectum	0.88	0.87-0.89	Rectum	0.88	0.87-0.89
Primary procedure	Major resection	1.00		Major resection	1.00	
	Local excision	1.68	1.62-1.75	Local excision	1.68	1.62-1.75

	Bypass	2.84	2.65-3.03	Bypass	2.84	2.65-3.03
	Stoma	2.27	2.21-2.34	Stoma	2.27	2.21-2.34
	Stent	1.85	1.76-1.94	Stent	1.85	1.76-1.94
	No surgical procedure	2.25	2.22-2.29	No surgical procedure	2.26	2.22-2.30
Admission method	Elective	1.00		Elective	1.00	
	Emergency	1.95	1.93-1.98	Emergency	1.95	1.93-1.98
Screening status	Symptomatic	1.00		Symptomatic	1.00	
	Screen-detected	0.46	0.40-0.53	Screen-detected	0.46	0.40-0.53
Year	2001	1.00		2001	1.00	
	2002	0.97	0.95-1.00	2002	0.97	0.94-0.99
	2003	0.95	0.93-0.98	2003	0.94	0.92-0.97
	2004	0.91	0.89-0.94	2004	0.91	0.89-0.93
	2005	0.91	0.89-0.94	2005	0.91	0.89-0.94
	2006	0.89	0.87-0.91	2006	0.89	0.87-0.91
	2007	0.88	0.86-0.90	2007	0.88	0.85-0.90
	2008	0.86	0.84-0.88	2008	0.85	0.83-0.88
Annual trust workload	Low	1.00		Low	1.00	
	Medium	1.00	0.98-1.01	Medium	0.99	0.98-1.01
	High	1.00	0.98-1.02	High	0.99	0.98-1.01
Trust ECMC status**	No	1.00		No	1.00	
	Yes	0.93	0.91-0.95	Yes	0.95	0.93-0.97

*The models have been adjusted for all factors listed in the table. Some of the covariate estimates appear to be identical when shown here to two decimal places but are different when looked at in more detail.
**The data were not materially altered whether or not patients managed within the hospital which lost its ECMC status in 2012 were included.

Supplementary Table 4: Association between high intervention trials research participation for each separate calendar year and five-year survival

	≥16% participation		≥7% participation	
**Year**	**HR[*]**	**95% CI**	**HR[*]**	**95% CI**
2001[a]	-	-	-	-
2002	0.97	0.92-1.01	0.99	0.96-1.03
2003	0.99	0.95-1.04	1.00	0.97-1.04
2004	0.90	0.82-0.99	0.98	0.94-1.02
2005[b]	1.09	0.97-1.23	0.98	0.93-1.03
2006	0.87	0.82-0.92	0.96	0.92-1.00
2007	0.91	0.85-0.97	0.95	0.92-0.99
2008	0.84	0.77-0.92	0.97	0.93-1.02

[*]Adjusted for age, sex, deprivation, stage, site, primary procedure, admission method, screening status, trust workload, ECMC status

[a]No trusts had high research activity in 2001 (≥16% or ≥7%)

[b]The point estimate for HR is high in 2005 with wide confidence limits and is not significant (p>.05). This is likely to be a chance finding since recruitment into interventional trials was low in 2005, with also an unusually low (2%) of trusts achieving >16% participation, amounting to 533 patients (0.2% of the total population), and these patients fared poorly, although the 2779 patients treated in Trusts which achieved 7-16% participation fared well in 2005, as in other years.

Supplementary Table 5: Multivariable analysis of the association between intervention trials research participation and 30-day post-operative mortality using a model-derived cut-point – Full results of the threshold and duration analyses

	Participation threshold (≥16% in any year)			Number of years with high participation		
	Adjusted 30-day mortality*			Adjusted 30-day mortality*		
**Variable**		**OR**	**95% CI**		**OR**	**95% CI**
Research participation	Low (<16%)	1.00		0 years	1.00	
	High (≥16%)	0.85	0.78-0.94	1 year	0.95	0.89-1.02
				2 years	0.93	0.85-1.02
				3 years	0.87	0.76-0.99
				4 years	0.76	0.67-0.86
Age group	<60 years	1.00		<60 years	1.00	
	60-70 years	2.27	2.04-2.52	60-70 years	2.26	2.03-2.52
	70-80 years	4.81	4.36-5.30	70-80 years	4.81	4.36-5.30
	>80 years	9.82	8.90-10.84	>80 years	9.83	8.90-10.85
Sex	Male	1.00		Male	1.00	
	Female	0.75	0.71-0.78	Female	0.75	0.71-0.78
Deprivation quintile	1 (least deprived)	1.00		1 (least deprived)	1.00	
	2	1.08	1.00-1.16	2	1.08	1.00-1.16
	3	1.12	1.04-1.21	3	1.12	1.04-1.21
	4	1.24	1.15-1.33	4	1.24	1.15-1.33
	5 (most deprived)	1.39	1.29-1.50	5 (most deprived)	1.39	1.29-1.50
Dukes' stage	A	1.00		A	1.00	
	B	1.12	1.03-1.23	B	1.12	1.03-1.23
	C	1.44	1.32-1.58	C	1.44	1.31-1.58
	D	2.04	1.83-2.28	D	2.05	1.84-2.28
Tumour site	Colon	1.00		Colon	1.00	
	Rectum	1.14	1.07-1.21	Rectum	1.14	1.07-1.21
Admission method	Elective	1.00		Elective	1.00	

	Emergency	4.00	3.81-4.20	Emergency	4.00	3.81-4.20
Screening status	Symptomatic	1.00		Symptomatic	1.00	
	Screen-detected	0.51	0.32-0.81	Screen-detected	0.51	0.32-0.82
Year	2001	1.00		2001	1.00	
	2002	1.00	0.91-1.10	2002	0.98	0.89-1.07
	2003	0.98	0.89-1.08	2003	0.96	0.87-1.05
	2004	0.97	0.89-1.06	2004	0.97	0.88-1.06
	2005	0.87	0.79-0.95	2005	0.87	0.79-0.95
	2006	0.89	0.81-0.98	2006	0.88	0.80-0.96
	2007	0.83	0.75-0.91	2007	0.82	0.74-0.90
	2008	0.77	0.70-0.85	2008	0.77	0.70-0.85
Annual trust workload	Low	1.00		Low	1.00	
	Medium	0.91	0.86-0.96	Medium	0.91	0.86-0.96
	High	0.91	0.87-0.97	High	0.91	0.86-0.97
Trust ECMC status**	No	1.00		No	1.00	
	Yes	0.86	0.80-0.93	Yes	0.90	0.83-0.97

*The models have been adjusted for all factors listed in the table. Some of the covariate estimates appear to be identical when shown here to two decimal places but are different when looked at in more detail.

**The data were not materially altered whether or not patients managed within the hospital which lost its ECMC status in 2012 were included.

Supplementrary Table 6: Comparison of the complete case and imputed multivariable analyses of five-year survival and 30-day post-operative mortality (low vs. high research participation)

	Five-year survival*			
	**Complete case**		**Imputed data**	
**Research participation**	**HR**	**95% CI**	**HR**	**95% CI**
Low (<16%)	1.00		1.00	
High (≥16%)	0.95	0.92-0.97	0.95	0.92-0.97
	**30-day mortality****			
	**Complete case**		**Imputed data**	
**Research participation**	**OR**	**95% CI**	**OR**	**95% CI**
Low (<16%)	1.00		1.00	
High (≥16%)	0.88	0.80-0.97	0.85	0.78-0.94

*Adjusted for age group, sex, deprivation quintile, Dukes' stage, tumour site, primary procedure, admission method, screening status, year of diagnosis, annual Trust workload, ECMC status.

**Adjusted for age group, sex, deprivation quintile, Dukes' stage, tumour site, admission method, screening status, year of diagnosis, annual Trust workload, ECMC status.

Supplementary Table 7: Multivariable analysis of the association between intervention trials research participation and one-year survival using an optimal cut-point approach

**Participation threshold (≥16% in any year)**			**Number of years with high participation**		
**Adjusted one-year survival***			**Adjusted one-year survival***		
	**HR**	**95% CI**		**HR**	**95% CI**
Low (<16%)	1.00		0 years	1.00	
High (≥16%)	0.95	0.92-0.98	1 year	0.99	0.96-1.01
			2 years	1.04	1.00-1.07
			3 years	0.91	0.87-0.95
			≥4 years	0.89	0.86-0.92

*Adjusted for age group, sex, deprivation quintile, Dukes' stage, tumour site, primary procedure, admission method, screening status, year of diagnosis, annual Trust workload, ECMC status.

Supplementary Figure

Supplementary Figure 1: The impact of the cut point approach on statistical power

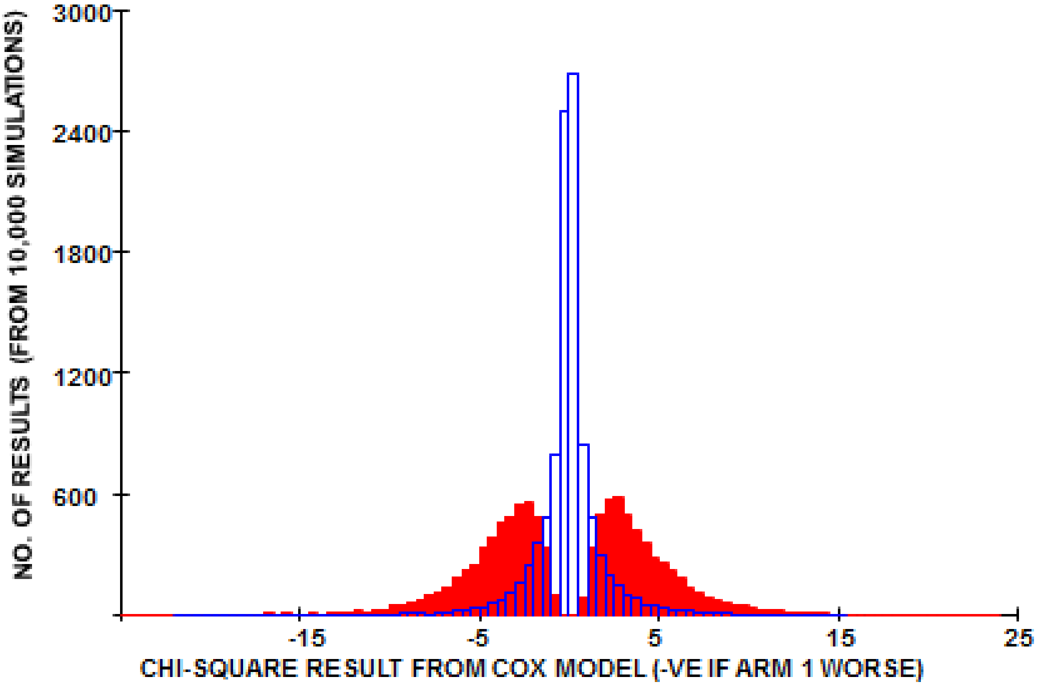

Suuplementary Figure 1 shows the distribution of null-hypothesis chi-square cox model results comparing optimum cut-point approach (red bars) with treating the variable as continuous (blue bars).
